# Learning health systems in primary care: a systematic scoping review

**DOI:** 10.1186/s12875-021-01483-z

**Published:** 2021-06-23

**Authors:** Danielle M. Nash, Zohra Bhimani, Jennifer Rayner, Merrick Zwarenstein

**Affiliations:** 1 Department of Epidemiology and Biostatistics, Western University, London, ON Canada; 2 ICES, London, ON Canada; 3 Department of Medicine, London Health Sciences Centre, London, ON Canada; 4 Centre for Studies in Family Medicine, Western University, London, ON Canada; 5 Department of Research and Evaluation, Alliance for Healthier Communities, Toronto, ON Canada; 6 ICES, Toronto, ON Canada

**Keywords:** Learning health systems, Primary care, Family medicine, Systematic review, Scoping review, Health systems improvement, Health systems research, Healthcare delivery, Quality of care

## Abstract

**Background:**

Learning health systems have been gaining traction over the past decade. The purpose of this study was to understand the spread of learning health systems in primary care, including where they have been implemented, how they are operating, and potential challenges and solutions.

**Methods:**

We completed a scoping review by systematically searching OVID Medline®, Embase®, IEEE Xplore®, and reviewing specific journals from 2007 to 2020. We also completed a Google search to identify gray literature.

**Results:**

We reviewed 1924 articles through our database search and 51 articles from other sources, from which we identified 21 unique learning health systems based on 62 data sources. Only one of these learning health systems was implemented exclusively in a primary care setting, where all others were integrated health systems or networks that also included other care settings. Eighteen of the 21 were in the United States. Examples of how these learning health systems were being used included real-time clinical surveillance, quality improvement initiatives, pragmatic trials at the point of care, and decision support. Many challenges and potential solutions were identified regarding data, sustainability, promoting a learning culture, prioritization processes, involvement of community, and balancing quality improvement versus research.

**Conclusions:**

We identified 21 learning health systems, which all appear at an early stage of development, and only one was primary care only. We summarized and provided examples of integrated health systems and data networks that can be considered early models in the growing global movement to advance learning health systems in primary care.

**Supplementary Information:**

The online version contains supplementary material available at 10.1186/s12875-021-01483-z.

## Background

Health system improvement in primary care is generally slow, partly because of the dependence on passive knowledge dissemination, but also because of the lack of a systematic approach to identify gaps between evidence and practice, and implementation of interventions to close these gaps [1, 2]. The spread of electronic health records (EHR) in primary care across North America in the past decade [3–5], and the ability to rapidly analyze these data and act on their findings, provides a strategy to potentially close the loop between practice gaps and implementation strategies. In 2007, the Institute of Medicine (IOM) first described the concept of a learning health system (LHS) [6], which is an organization or a network with a culture of health system improvement where internal data is integrated with existing evidence and rapidly analyzed, this knowledge is put into practice, and its effectiveness at closing practice gaps is evaluated [7].

Budrionis and Bellika (2016) completed the most recent systematic review on LHSs and found 13 papers that described implementation of an LHS [8]. They categorized these into clinical data reuse (nine), patient-reported outcomes (three), and collaborative learning (one). Most of these LHSs were in the hospital setting, and it’s not clear how many also included primary care [8]. There are no systematic or scoping reviews summarizing LHSs in primary care.

Primary care is a patient’s first point of contact with the healthcare system, and typically providers focus on primary and secondary prevention of chronic diseases and acute episodes. Many primary care providers now use EHRs [3–5], which can be used to track illness and healthcare patterns, provide decision support, and for quality improvement initiatives. On the other hand, many primary care providers work in small independent community practices and use one of various EHR vendors, making it difficult to link data across different practices, especially in Canada [9]. Therefore, the primary care setting is both an opportune yet challenging setting to develop an LHS.

The purpose of this scoping review was to understand the extent of LHSs’ spread in primary care and their characteristics. We decided to conduct a scoping review to capture a broad overview on this topic. Our research objectives were to understand: 1) where LHSs in primary care settings have been implemented or are being planned, 2) how these LHSs are operating, and 3) the challenges and solutions to implementing or sustaining an LHS in primary care.

## Methods

We completed a scoping review following guidelines outlined by the Joanna Briggs Institute and the PRISMA extension for scoping reviews (Additional file 1) [10, 11]. We do not have a published protocol for this study.

### Eligibility criteria

We included data sources that described the implementation or plans for developing an LHS within a primary care setting. We defined an LHS as an organization or a network, which rapidly analyzes health data while incorporating best-practice guidelines to directly feedback to and improve clinical care (either through clinical research, quality improvement or decision support tools). In this case, clinical research included comparative effectiveness analyses, prediction modelling, pragmatic trials, or other big data analytics where the findings could be readily applied in practice. We excluded research networks where only conventional knowledge translation through academic publications was occurring without more direct feedback of the knowledge to improve care. We included integrated health systems or networks that also had inpatient care, or care in other settings, if they included primary care in their health system or network. We did not include pediatric LHSs, since this is considered specialty care in some countries including Canada. We only included studies published after the IOM report in 2007 [6], and we restricted the search to English language articles only. Finally, we did not include sources describing only specific technical components of an LHS, such as EHRs, software, hardware or interoperability.

### Information sources

We searched OVID Medline®, Embase® and IEEE Xplore® from January 2007 to January 2020. We originally searched databases on March 24, 2019 and updated searches on January 2, 2020. We also reviewed all papers published in the following journals: *Leaning Health Systems*, *eGEMs*, and *BMJ Quality and Safety*. We then reviewed all references from Budrionis and Bellika (2016) and other systematic reviews that were found from our database search [8]. We also used Google Scholar to find articles that cited the IOM’s (2007) LHS report [6]. To capture gray literature we also performed a Google search in August 2019 for ‘learning health systems’. To confirm whether an LHS was eligible for inclusion in our review, we reached out to some authors or website contacts.

### Search strategy

We consulted a librarian at Western University on the search strategy (Additional file 2). For Medline® and Embase® we used keywords for different variations of LHSs including ‘health’ versus ‘health care’. We also included Medical Subject Headings (MeSH) terms for ‘learning’ and ‘health care delivery’. We used search terms for primary care in the IEEE Xplore® database search but not for the other databases, since this narrowed the articles retrieved and did not capture integrated health systems. After all eligible data sources were found we completed a targeted search using Pubmed and Google for other sources of information further describing these LHSs. This was to ensure completeness for our data charting. For example, an article found through our search may have discussed the LHS in detail but lacked information on the organization or health system in which it was based.

### Selection and data charting processes

All articles from the database search were uploaded to Covidence [12]. DMN screened all article titles and abstracts through Covidence and selected ones eligible for full-text review, and then completed the full-text review. ZB further reviewed full-text articles for eligibility, and both authors discussed discrepancies. DMN created the data charting form and tested it on five articles to ensure all information would be captured. ZB charted the data from the eligible articles and websites, and DMN reviewed and confirmed all information entered in the spreadsheet against the original data source. The charted data elements are described in Additional file 3. We summarized data in table format (overview of LHSs, and challenges and solutions) and included a description of each learning health system with examples of primary care specific learning initiatives in the text. In some cases, we reached out to authors or website contacts to further confirm details.

## Results

### Selection of sources of evidence

**Fig. 1:**
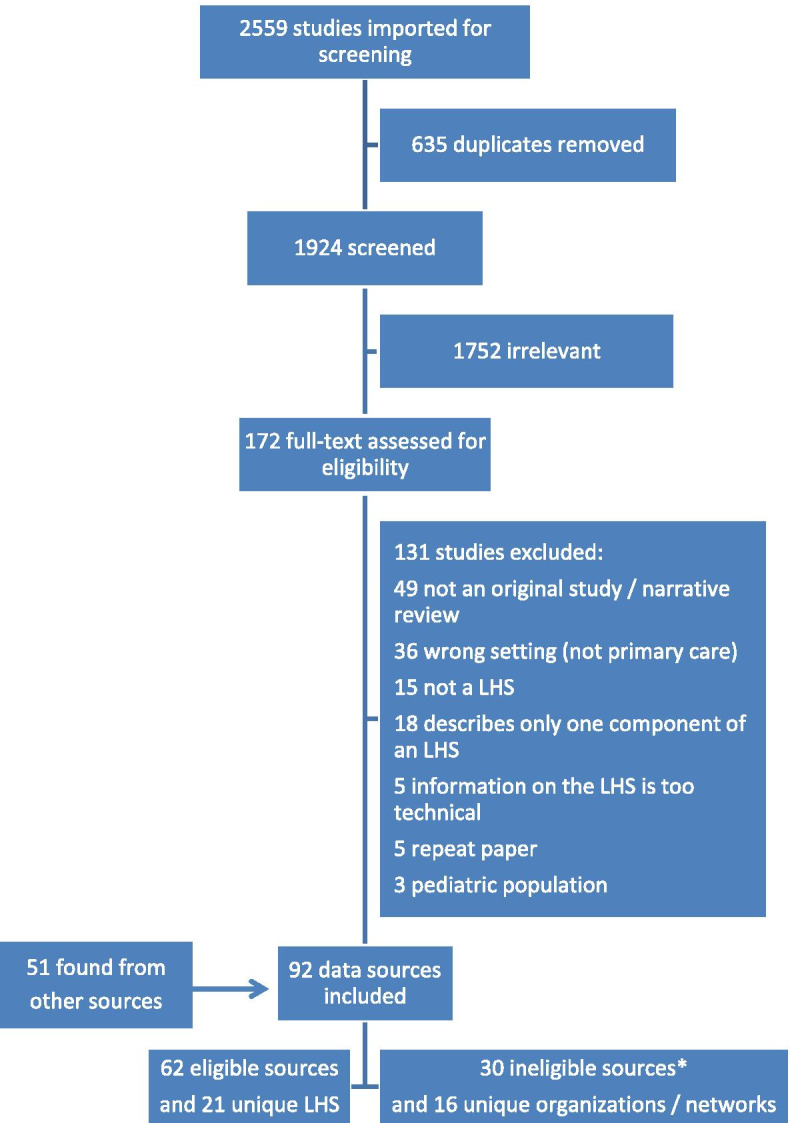
The number of data sources included and excluded in review, and reasons for exclusions. *Ineligible sources refer to data networks and organizations that did not meet our definition of a learning health system but were included as an additional file of those with potential to become one

Through our database search we retrieved 1924 unique articles plus an additional 51 from other sources. The number of articles excluded from the database screening, and the reasons for exclusion are shown in Fig. 1. Confirmation of eligibility by reaching out to authors confirmed inclusion for one data source and confirmed exclusion for two. We only searched up to page 8 in Google for our internet search, since the results were not relevant after this.

### Characteristics of sources of evidence

We identified 21 LHSs including primary care that met our definition, from 40 peer-reviewed research articles and 22 websites (Table 1). Most (18/21) self-identified as an LHS, where three did not: Health Care Systems Research Network (HCSRN), High Value Healthcare Collaborative (HVHC), and Intermountain Healthcare [13–15].

**Table 1:** Overview of learning health systems included in the review

Name	Location	Description of Organization	Stage	Purpose
**Integrated health system**				
Baylor Scott & White Health [16]	Texas, U.S	Consists of 48 hospitals, 185 outpatient facilities, 26 surgery centers, 164 primary care clinics, 503 specialty clinics, and 30 pharmacies	Planning	Clinical decision making / quality improvement
Denver Health [17]	Colorado, U.S	Consists of a hospital, 17 school health clinics, 9 primary care centers, and a trauma center	Planning	Clinical decision making / quality improvement
Geisinger Health System [18–22]	Central Pennsylvania & Southern New Jersey, U.S	Consists of 8 hospitals, local primary care centers, specialists, and trauma centers	Active	Clinical decision making / quality improvement
Intermountain Healthcare [15, 23–25]	Utah & Idaho, U.S	Consists of 22 hospitals, over 179 clinics, physician offices, and home-based care centers	Active	Clinical decision making / quality improvement
Johns Hopkins [26]	Maryland, U.S	Consists of 240 primary care providers	Active	Clinical decision making / quality improvement
Kaiser Permanente Colorado [27, 28]	Colorado, U.S	Consists of 1200 physicians across 31 centers	Active	Research / clinical decision making
Kaiser Permanente Northern California [29–31]	Northern California, U.S	Consists of 8000 physicians across 237 centers	Active	Research / clinical decision making
Kaiser Permanente Southern California [32–35]	Southern California, U.S	Consists of 6000 physicians across 209 centers	Active	Research / clinical decision making / quality improvement
Kaiser Permanente Washington formerly known as Group Health Cooperative [36, 37]	Washington, U.S	Consists of 31 medical centers	Active	Clinical decision making / quality improvement
NYU Langone Health [38, 39]	New York, U.S	Consists of 6 inpatient locations, and 8 primary care and speciality clinics	Active	Clinical decision making / quality improvement
University of Utah [40]	Utah, U.S	An integrated health system consisting of 4 hospitals, 12 primary care clinics, 23 regional partners, and 3 specialty centers	Active	Quality improvement
University of Wisconsin [41, 42]	Wisconsin, U.S	An integrated health system with approximately 1500 physicians, and 45 primary care clinics with over 250,000 patients	Active	Clinical decision making / quality improvement
Veteran’s Affairs [43–52]	U.S	National network across the Veterans Health Administration consisting of over 1200 healthcare centers, including primary care	Active	Research / quality improvement
**Research / data network**				
Connected health cities [53, 54]	United Kingdom	A network of 4 cities in North England collaborating with many organizations including NHS, hospitals, universities, and 550 primary care provider centers	Completed	Research / quality improvement
Health Science South Carolina [55, 56]	South Carolina, U.S	A network consisting of 7 of South Carolina's largest health systems and 3 research universities with a central data warehouse	Active	Research / quality improvement
Northwestern University Clinical and Translational Sciences Institute (NUCATS) [57, 58]	Chicago, U.S	A network that supports clinical and translational research across Northwestern University departments, including clinical partners of Northwestern Memorial Healthcare Corporation, Ann and Robert H. Lurie Children's Hospital, and the Rehabilitation Institute of Chicago, and community partners including primary care clinics	Active	Research / quality improvement
Swiss Learning Health System (SLHS) [59, 60]	Lucerne, Switzerland	A network consisting of partner organizations: the University of Lucerne, University of Zurich, Swiss TPH Basel, Zurich University of Applied Sciences, University of Neuchâtel, University of Lugano, and University of Applied Sciences and Arts of Southern Switzerland	Planning	Research
Translational medicine and patient safety (TRANSFoRm) [61–68]	Europe	A network of 21 partner organisations from 10 EU member states focusing on primary care research	Active	Research / clinical decision making
**Network of networks**				
Healthcare Systems Research Network (HCSRN) [13, 69]	U.S	A network consisting of 19 healthcare systems across 13 states including Kaiser Permanente and Geisinger	Active	Research
High Value Healthcare Collaborative (HVHC) [14, 70, 71]	U.S	A network consisting of 19 healthcare delivery systems, including integrated systems with primary care, from across the U.S. with the data hub at the Dartmouth Institute for Health Policy and Clinical Practice	Active	Research
Optum Labs [72–74]	U.S	A network with headquarters in Cambridge, Massachusetts that standardizes EHR data across 52 health systems in the U.S	Active	Research

*Abbreviations*: *EHR* electronic health records

Translational medicine and patient safety (TRANSFoRm) was the only LHS included in our review that operated only in primary care [61]. The remaining LHSs are vertically integrated health systems or networks that deliver care through hospitals, universities and primary care. Although both are integrated health systems, the papers describing Johns Hopkins’ and University of Wisconsin’s LHSs focus on primary care. Three of the LHSs identified were in Europe [53, 59, 61], where the remaining were in the United States.

All but one of the included LHSs appear to be financially sustainable, with the learning activities from most organizations funded through care delivery, rather than external, project-based or time-limited funding. The exception is TRANSFoRm, which is a time-limited research project dependent on external funding [18, 61]. However, even internally-funded LHSs, such as Geisinger Health System, raised concerns about financial sustainability of their learning activities [75].

We also identified 16 additional data networks / organizations (based on 14 peer-reviewed research articles and 16 websites) that included primary care data, but did not meet our definition of a fully-functioning LHS, since they were mostly platforms for conventional research and knowledge translation, rather than building internal systems for directly identifying gaps and cycling knowledge to improve care (Additional file 4). The majority of these (12/16) were projects funded through the National Patient-Centered Clinical Research Network (PCORnet), with the initial aim to conduct large-scale comparative effectiveness research [76]. We included all 16 in an additional file as “potential LHSs”, rather than excluding them from our review, since many are working towards an LHS by establishing a data sharing process. However, our review and search strategy targeted LHSs, so the identified research / data networks and organizations may not be comprehensive.

### Integrated health systems

We identified 13 integrated LHSs in our review, all of which are in the United States.

Geisinger Health System – a large not-for-profit integrated health system in Central Pennsylvania and Southern New Jersey encompassing eight hospital campuses – was an early adopter of LHS approaches [19]. In Psek (2015), they reported a framework for nine components of an LHS and outlined how Geisinger meets these components including data and analytics, people and partnerships, patients and family engagement, ethics and oversight, evaluation and methodology, funding, organization, prioritization, and deliverables [20]. We did not find any documented primary care-specific LHS initiatives for Geisinger.

Kaiser Permanente is another large not-for-profit integrated health system serving members across eight states and the District of Columbia [77]. We identified many examples of LHS activities among the different Kaiser Permanente sites [29, 32]. Kaiser Permanente Southern California developed an Outpatient Safety Net Program, which uses clinical surveillance software to regularly scan outpatient EHR data to identify care gaps [33]. Similar initiatives included screening for new diagnoses and ensuring follow-up on positive tests, such as cancer screening, kidney disease, and Hepatitis C, and identification of potentially harmful medications and interactions, or those that require laboratory monitoring.

To address an identified problem of missed appointments, Kaiser Permanente Colorado investigators conducted a randomized clinical trial and showed that phone call and text message based reminders to patients significantly reduced missed appointments [27]. They also developed and externally validated a prediction model to identify those most likely to miss their appointment.

The Veterans Health Administration (VHA) is the largest integrated health system in the United States consisting of a national network with more than 1200 centers (including over 1000 outpatient clinics) serving 9 million veterans [43]. The VHA uses a combination of research and quality improvement to improve care [44, 45]. They demonstrated one way to embed research into clinical practice through an initiative called Point of Care Research (POC-R) [46]. This initiative facilitates pragmatic trials using EHR data by fully embedding these trials into practice. For example, when a patient is eligible for one of the ongoing trials, a notification is sent through the EHR asking the clinician if they want to randomize the patient or not. A research coordinator then obtains patient consent and all data is collected through the EHR.

Another VHA initiative called Quality Enhancement Research Initiative (QUERI) is an internally-funded program to help the VHA translate research into practice more quickly and efficiently than traditional knowledge translation [45, 47, 48]. Four priority research projects were: 1) home- and community-based care for Veterans at risk of nursing home placement, 2) risk mitigation for patients receiving opioid prescriptions, 3) targeting care for patients at high risk for suicide, and 4) a telehealth tool to improve access to dermatology services. An Implementation Roadmap was also developed to scale up these initiatives and to help clinicians sustain them in practice [48]. This Roadmap was developed based on a review of existing frameworks and successful implementation strategies and based on the expertise of QUERI programs. However, further research and evaluation is needed to confirm and further refine Roadmap components [48].

Intermountain Healthcare is a not-for-profit integrated health system serving patients primarily in Utah, southern Idaho, and southern Nevada [23]. Intermountain Healthcare was an early adopter of a data warehouse in 1998 to measure best practices and costs, and to inform quality improvement initiatives [15]. They continue to use their data to identify gaps and quality improvement efforts to inform clinical decision making [24]. One example is the development of an Area Deprivation Index (a proxy for socioeconomic status), which was evaluated in a prediction model and found to help identify those who would benefit most from enhanced care management services [78].

At Johns Hopkins there are 240 primary care physicians serving over 250,000 patients across Maryland [26]. McGuire (2019) describes the evolution of developing an LHS within primary care at Johns Hopkins, including clinician experts and a quality analyst who provides EHR support, development of a team-based learning culture among staff and clinicians, improvement of patient experience, and incentives for participation in learning activities among clinicians [26]. We could not identify any documented specific LHS projects for Johns Hopkins.

The University of Wisconsin developed a framework for learning health system development and sustainability through their Health Innovation Program, which focuses on research being an equal component to health care [41]. One example using this Health Innovation Program framework is the development of a health case management program to improve primary care for patients with complex medical or social care considerations. Through this program, 20 nurses and social workers were hired as case managers to provide telephone support to these patients. They also developed a prediction model to identify patients who would benefit most from this program. This health case management program is being scaled up across all their primary care clinics, and future evaluations will assess the impact of this scale up [41].

NYU Langone Health has six hospitals and eight primary care and specialty centers [38]. They implemented a rapid-cycle initiative to evaluate existing practices in randomized trials to determine whether they should continue or be modified, and to eventually test new quality improvement initiatives. Over a one-year period, ten existing delivery practices were evaluated – most within primary care – including prompts for flu vaccines and smoking cessation, mailed reminders for appointments, comparison of different telephone scripts for annual visits, and messages to patients to complete health surveys [39].

The Agency for Healthcare Research and Quality (AHRQ) in the United States highlighted the following three large integrated healthcare organizations on their journeys to becoming LHSs: 1) Baylor Scott and White Health – the largest not-for-profit health system in Texas, 2) Denver Health – providing comprehensive care to a third of all Denver residents, and 3) University of Utah Health – an academic healthcare system in Salt Lake City, Utah [16, 17, 40]. Baylor Scott and White Health is planning to transition all their sites to a single EHR and create value-based dashboards for all primary care physicians to standardize information [16]. Denver Health has launched a Quality Improvement Review Committee to review proposals for new initiatives [17]. Denver Health and University of Utah are both using their EHRs to benchmark progress in comparison to baseline data and other leading institutions [17, 40]. University of Utah Health is also using monitoring systems to provide real-time feedback on system-wide issues [40]. However, all three organizations do not seem to have used internal data systems to evaluate the impact on patient care, outcomes or costs of these changes to their practices.

### Research / data networks and networks of networks

TRANSFoRm is a network of 21 partner organizations from 10 European countries focusing on primary care research [61]. TRANSFoRm was originally developed for three purposes: 1) prospective study recruitment including randomized trials, 2) retrospective analyses, and 3) decision support for clinical care [62, 63]. Through TRANSFoRm, an electronic solution was developed and validated for standardized and automatic recruitment and data collection for pragmatic clinical trials embedded in EHRs, which is now being deployed in the United Kingdom [79]. The decision support component focused on embedding diagnostic support for primary care clinicians within their EHRs advising on earlier cancer diagnoses [64]. An expansion of TRANSFoRm includes ROAD2H, which is an LHS that will provide decision support combined with local clinical guidelines for primary care to low- and middle-income countries [65].

We identified two other learning health data networks also in Europe: the Swiss Learning Health System (SLHS) in Lucerne, Switzerland and Connected Health Cities in North England, United Kingdom [53, 54, 59]. Projects are being advanced within the SLHS including themes of innovation in service delivery, health promotion and prevention, and health systems guidance and intelligence [60]. The Connected Health Cities was a pilot project leading to over 10 million citizens across North England with connected health records [53]. Sixteen clinical pathway projects have been developed using these health records to help improve patient care. One example is the development of a National Antibiotic Prescribing Dashboard, which uses anonymous data to allow primary care physicians to compare their antibiotic prescribing practices to national and local averages, and allows them to identify higher risk patients [53].

In the United States, we identified two university-based learning health networks that include primary care: Northwestern University Clinical and Translational Sciences Institute (NUCATS) and Health Sciences South Carolina [55, 57]. NUCATS supports clinical trials, community-based research, as well as dissemination and implementation of findings [57]. We could not identify any documented primary care-specific LHS initiatives for NUCATS and Health Sciences South Carolina.

We identified three network of networks, where two of these – the Healthcare Systems Research Network (HCSRN) and the High Value Healthcare Collaborative (HVHC) – each consists of 19 different healthcare systems across the United States, and Optum Labs which standardizes EHR data from across 52 health systems in the United States [13, 14, 72]. Optum Labs describes the ability to use their healthcare data to conduct “N of 1” studies, so healthcare providers can generate evidence that is directly applicable to complex, unique patients at the point of care [72]. We could not identify any documented system level improvement activities nor any primary care-specific LHS initiatives for HCSRN and HVHC.

### Challenges and potential solutions

The LHSs included in this review identified challenges that they experienced or anticipated and potential solutions to these challenges in regards to data standardization or quality, ease of data access and use, financial sustainability, promoting a culture of learning, involvement of patients and the community, consistency across different sites within the same organization, prioritization of learning initiatives, the use of EHRs for quality improvement, and the move toward embedding evaluation and implementation of improved practices into usual care rather than traditional research projects (Table 2).

**Table 2:** Summary of challenges and potential solutions identified by the included learning health systems

Challenges	Potential Solutions
**Data**	
Lack of standardized data or data that is low quality or missing [46, 55].	Use of a ‘mediation’ approach to data interoperability (i.e. standardization) allows for different EHR vendors to be linked, saves time and money from reorganizing the whole network, allows new data to easily be incorporated, and for flexibility with how the data is used [63]. Adapting interventions to fit existing EHRs to ease dissemination of findings [30]. Standardization of data and processes across systems [13].
Lag in updated data including patient lists [15].	Real-time access to and analysis of data [17, 20, 40, 70].
Need to access data without the assistance of a data analyst [21].	Use of LHS tools / dashboards with minimal or no training (i.e. ease of use by all providers) [67, 80].
Patients who do not get all their care through one system / organization or out of pocket expenses not covered, so complete data is not captured [15, 27, 45, 70].	Universal healthcare coverage for some regions including Switzerland and the United Kingdom [53, 59].
**Organizational factors**	
Uncertainty of financial sustainability [18, 22]	Internal drivers and resources within the organization, rather than depending on external funding [13, 53, 57, 73].
Increasing awareness of LHS and developing a culture of learning and improving throughout organization [21].	Broad adoption of programs across leadership and providers [24, 35]. Embedding researchers within the healthcare system [13]. Training highly qualified personnel and educating healthcare providers to support a sustainable culture for learning and encouraging participation in learning / quality improvement activities [16, 17, 34, 40].
Need to involve patients and community [20, 21, 31, 34].	Initiatives to increase patient and family involvement, including the development of patient and family advisory councils, or allowing patients to access their data through secure patient portals [20].
Need to reduce practice variation across different sites [24, 44].	Use of better tools to reduce practice variability, including strategies to engage and help low-performing practices [44].
Need to develop processes to assist in prioritizing learning across the organization [20].	Priority setting within the organization to identify high-impact projects and initiatives, including the development of a committee to review and approve proposals [16, 17, 39, 40].
**Research / quality improvement**	
Need to make current EHR systems work better for research or quality improvement / how to deal with the extra time required by providers to participate [30, 46].	Incorporating data collection for research or quality improvement into clinical care rather than it being extra work for providers or staff [46, 51].
Need for quality improvement activities rather than traditional research to allow for more efficient analyses that can be easily incorporated into practice [44, 70].	Different initiatives to support quality improvement across the organization. For example: ○Holding annual quality improvement conferences where project leaders can share their experiences, which are then collated to provide a library of quality improvement initiatives [16]. ○A whiteboard in each clinic that lists all system-wide and clinic-specific quality improvement initiatives currently in progress at that location [17].

*Abbreviations*: *EHR* electronic health records, *LHS* learning health system

## Discussion

### Summary of evidence

Large integrated health systems in the United States and research / data networks in the United States and Europe provide some of the leading examples on developing an LHS within primary care. We identified only one LHS that operates exclusively in primary care, which is a research-funded initiative in Europe, TRANSFoRm. It is not clear from the data sources how integrated TRANSFoRm is with healthcare delivery organizations, since their partners are primarily academic organizations [62–65, 79]. The PCORnet projects demonstrate the United States’ motivation to move towards a national-level LHS, yet many of these networks need to apply more rapid quality improvement initiatives to keep moving in this direction rather than relying on traditional research [76, 81].

We identified challenges to initiating or sustaining an LHS in primary care and some potential solutions to these challenges. However, these solutions were presented at a high level and may need to be described in more detail to be useful for other organizations and networks looking to apply them.

### Implications and recommendations

Integrated health systems are at an advantage of having access to large amounts of healthcare data and delivering care to patients, compared to organizations that have access to healthcare data but are not directly responsible for delivering care. Furthermore, learning activities are generally funded by patient care revenue, so savings or improvements can be directly applied, thus providing an ideal platform for a self-sustaining LHS.

On the other hand, data networks develop the mechanisms and find the resources to link and analyze healthcare data, and although the data contributors to the network may be healthcare organizations, the networks themselves do not provide patient care, making it difficult for networks to directly develop interventions to improve care across all member organizations. This was why many of the data networks identified in this review did not meet our definition of a fully-functioning LHS. The data networks that were eligible provided some examples of using data to improve healthcare or at least plans for how they will become a fully-functioning LHS, but they generally showed less maturity than the integrated health systems. Network of networks, such as Optum Labs [72, 73], also use a different approach than the integrated health systems, which has some advantages and disadvantages. They provide a service for data integration and analysis, and a platform for collaboration and research. Although initial investments are required to establish these networks, they could become financially sustainable through the researchers and organizations who pay for their services. Similar to data networks, a disadvantage is their disconnect between healthcare provision and thus their limited ability to directly impact patient care at the system level, as compared to integrated health systems. For both types of networks, the separation between data analysis and responsibility for care improvement may limit their ability to design and evaluate effective interventions to improve care, along the same lines that integrated health systems have achieved.

The purpose of this review was to describe LHSs in primary care, however, we found most of the identified LHSs included other healthcare settings. This could be an advantage, since integrated organizations can apply learnings to the appropriate level more easily than primary care providers who are not closely linked to the rest of the system. Furthermore, many of the learning initiatives described included specific projects and not overall plans for whole system improvement in primary care. Geisinger is one of the longest established self-identified LHSs, and not surprisingly describes how their organization met all the criteria for an LHS, but even here there is little information on how this extends to the primary care setting [20]. Finally, the learning initiatives described in this review mainly include sequential research projects or quality improvement activities, but for an LHS to be as productive as possible it needs to move beyond sequential projects to continual improvement without sacrificing quality. NYU Langone Health seems to be moving in this direction with their rapid-cycle testing of various healthcare interventions using randomized clinical trials [39].

### Limitations

There are some limitations of our review that should be noted. We used keywords for LHSs to identify eligible studies. However, this may have biased our review to identifying more LHSs in the United States where this concept is more established, rather than other parts of the world where organizations may not realize that they are functioning as an LHS. It was beyond the scope of this review to seek out LHSs that were not self-identifying as such, although we did find a few using our search strategy. This presented another challenge of identifying organizations and networks that are true LHSs that go beyond just a data network, as there is not a clear definition of an LHS in the literature that can be easily applied to assess organizations as such. We created a definition based on existing literature and then refined this through the current search as we identified organizations that exemplified this criteria. We recognize that our definition may be a simplification of the concept. For example, Geisinger identified nine criteria that are required for an LHS [20]. However, most of the identified data sources did not provide enough details to assess whether or not they met all nine criteria, so if we applied this definition we would have underestimated the LHSs included in our review. Furthermore, LHSs also exist on a smaller scale, including solo-practicing physicians who use their data to learn and provide better care, but this was beyond the scope of this review.

We restricted our searches to English language only, which may also have biased the review towards English-speaking countries such as the United States and the United Kingdom. Finally, we only had one individual available at the time to perform the title and abstract review, even though guidelines recommend that two people should be involved at this step.

## Conclusions

This is the first scoping review to identify the extent and characteristics of LHSs in primary care. We identified 21 LHSs that included primary care, although all but one included care from other settings, and most were in the United States. We presented some example projects and some challenges and potential solutions that can be applied to future primary care LHSs. The potential of LHSs in primary care has started to be realized and will hopefully be expanded on in the future as more data networks and organizations move toward an LHS and focus on quality improvement that goes beyond traditional research.

## Supplementary Information

**Additional file 1**. Preferred Reporting Items for Systematic reviews and Meta-Analyses extension for Scoping Reviews (PRISMA-ScR) Checklist. Reporting checklist for systematic reviews

**Additional file 2**. Search strategies for Medline®, Embase® and IEEE Xplore®. Screenshots of the searches completed through different databases, including the keywords and mesh terms used

**Additional file 3**. Charted data elements and definitions or scenarios for meeting certain criteria. List of charted data elements abstracted from the included articles and definitions or scenarios for meeting certain criteria

**Additional file 4**. Integrated health systems and data networks with potential to become learning health systems. Summary of the identified integrated health systems and data networks with potential to become learning health systems and explanation for why they were not considered fully-functioning learning health systems

## Data Availability

The datasets used and/or analysed during the current study are available from the corresponding author on reasonable request.

## Abbreviations

AHRQ: Agency for Healthcare Research and Quality
EHRs: Electronic Health Records
HCSRN: Health Care Systems Research Network
HVHC: High Value Healthcare Collaborative
IOM: Institute of Medicine
LHS: Learning Health System
NUCATS: Northwestern University Clinical and Translational Sciences Institute
PCORnet: Patient-Centered Clinical Research Network
POC-R: Point of Care Research
QUERI: Quality Enhancement Research Initiative
SLHS: Swiss Learning Health System
VHA: Veterans Health Administration
TRANSFoRm: Translational medicine and patient safety
