# Supplementary material for: Learning health systems in primary care: a systematic scoping review
Source: BMC Fam Pract. 2021 Jun 23;22:126. doi: 10.1186/s12875-021-01483-z (PMC8223335; doi:10.1186/s12875-021-01483-z)

**Additional File 2. Search strategies for Medline**®**, Embase**® **and IEEE Xplore**®

OVID Medline® 1946 onwards


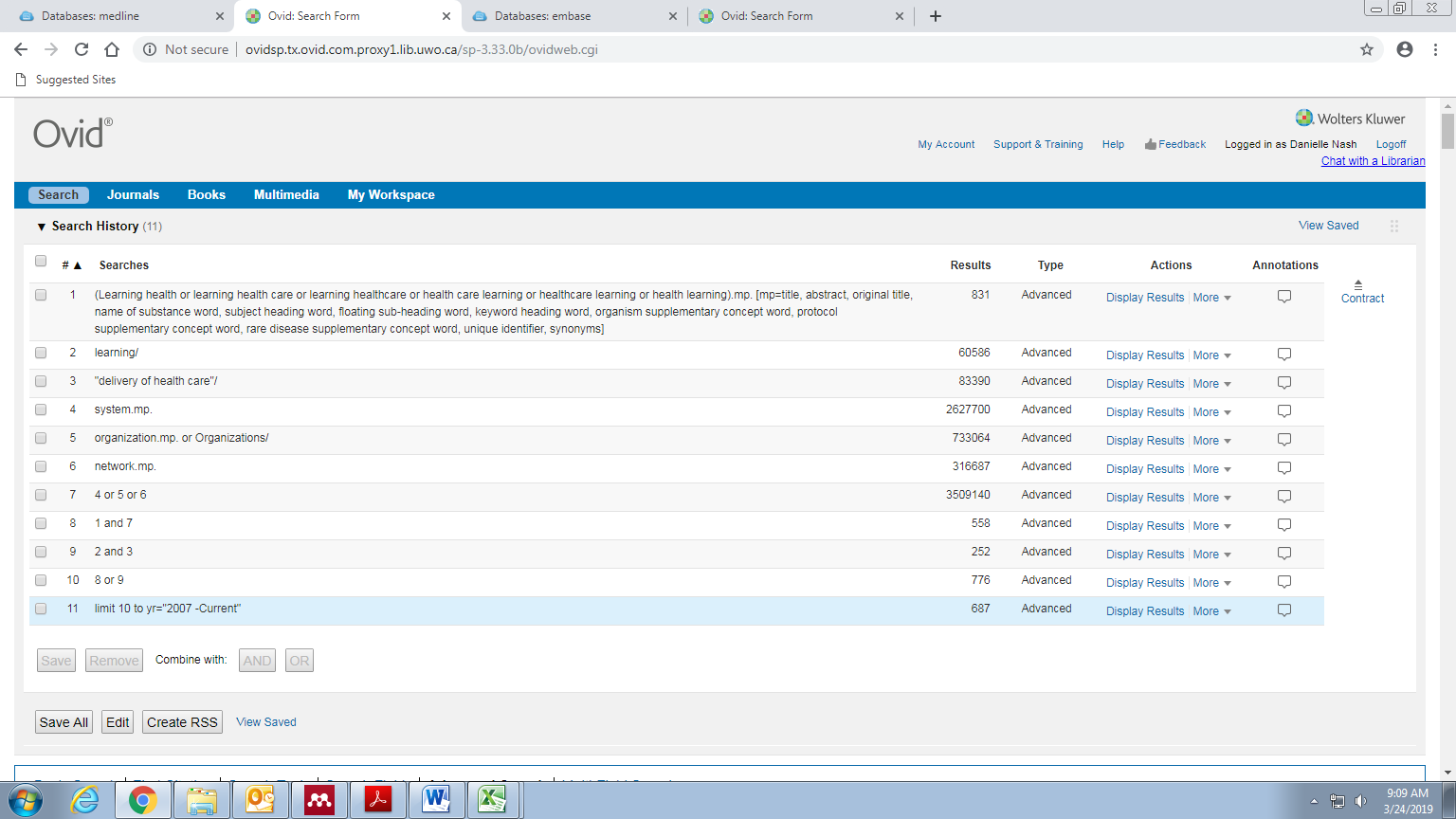


OVID Embase® from 1947 onwards


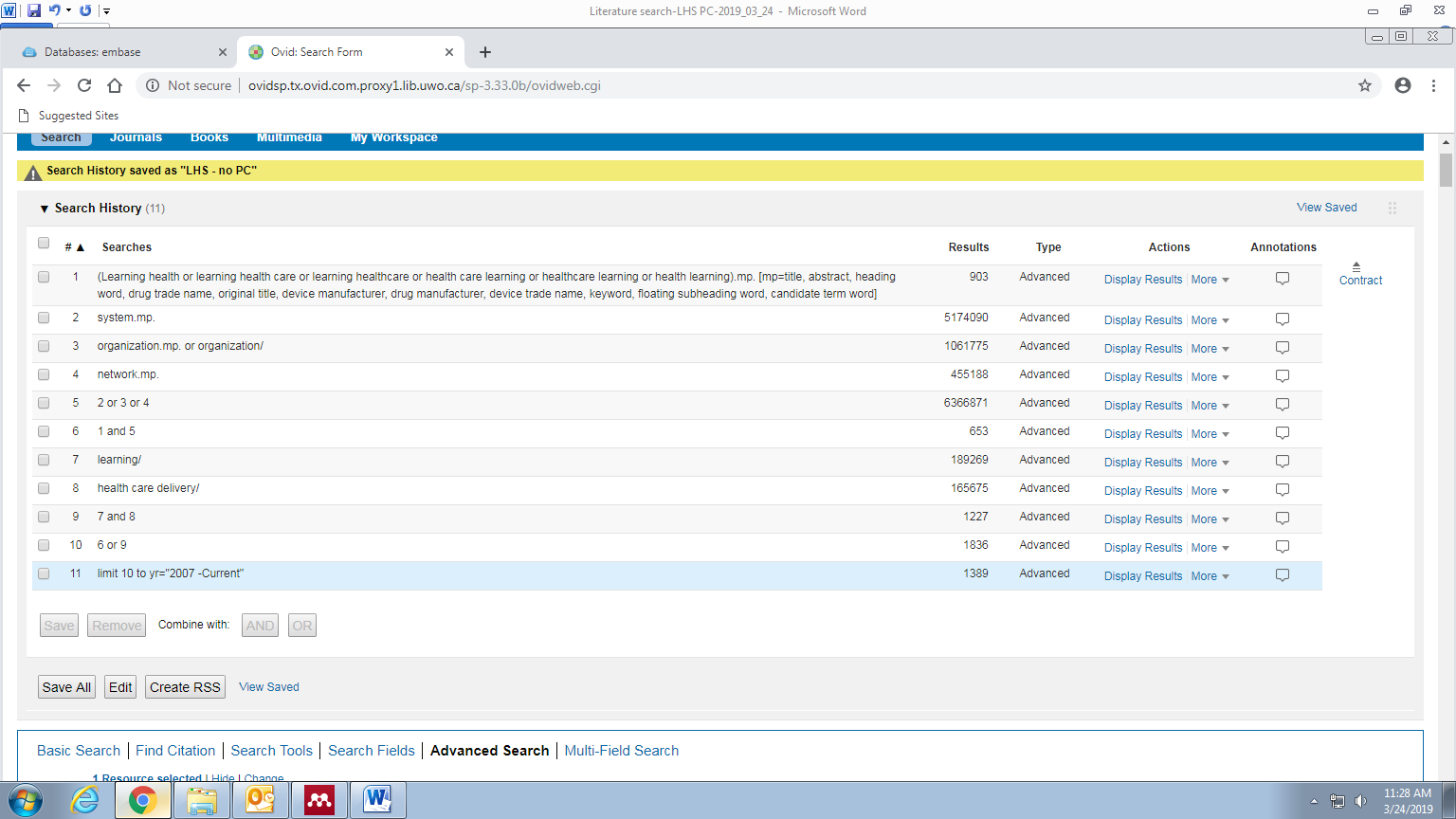


IEEE Xplore®


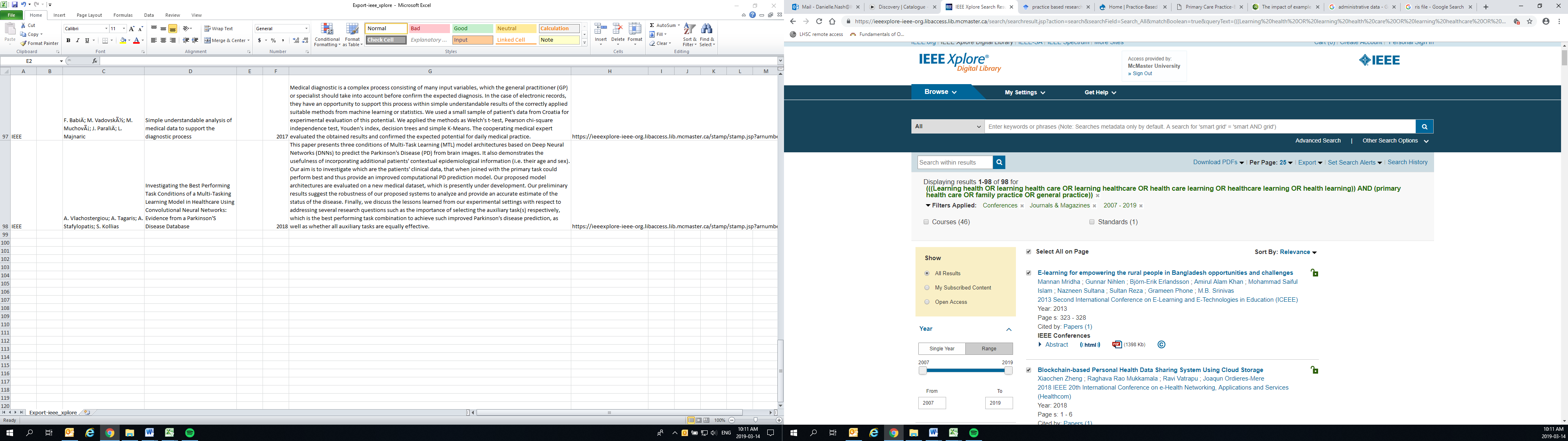

Supplement: Supplementary file 2 — Additional file 2. Search strategies for Medline®, Embase® and IEEE Xplore®. Screenshots of the searches completed through different databases, including the keywords and mesh terms used [file 12875_2021_1483_MOESM2_ESM.docx]
