# Supplementary material for: Learning health systems in primary care: a systematic scoping review
Source: BMC Fam Pract. 2021 Jun 23;22:126. doi: 10.1186/s12875-021-01483-z (PMC8223335; doi:10.1186/s12875-021-01483-z)
Supplement: Supplementary file 3 — Additional file 3. Charted data elements and definitions or scenarios for meeting certain criteria. List of charted data elements abstracted from the included articles and definitions or scenarios for meeting certain criteria [file 12875_2021_1483_MOESM3_ESM.docx]

**Additional File 3. Charted data elements and definitions or scenarios for meeting certain criteria**

| **Data Element** | **Description** |
| --- | --- |
| **Description of learning health system** | LHS or organization name; Description and; Location |
| **Structure of the learning health system** | **Integrated health system:** call themselves an integrated health system or consist of multiple locations and settings (e.g., inpatient and community-based care) which serve the same patients and are connected in a meaningful way (e.g., same EHR, insurance plan, etc.).  **Data / research network:** includes more than one healthcare organization or health system but they are not naturally connected to each other, rather they are connected through a central data hub / data coordinating center, or the data itself is not integrated but they are connected through the use of shared resources and governance.  **Network of networks:** similar to a data network but contains more than one integrated health system or data network that already have multiple connected health systems / organizations. |
| **Purpose of the learning health system** | **Research*:** describes the initiatives or use of the data as research and there is evidence of this (e.g., require ethics approval, recruitment of participants, publishing of research papers).  **Quality improvement*:** describes the initiatives or use of the data as quality improvement and there is evidence of this (e.g., does not require ethics approvals, makes use of patient data in the EHR / health system rather than collecting additional data from patients, does not result in publication of findings, the initiative is evaluated and may be scaled up within the organization / network).  **Clinical decision making*:** describes using tools or applying algorithms within their health system (generally within the EHR) to assist with clinical decision making at the point of care; these initiatives may or may not have been tested as either research or quality improvement.  *These are not discrete categories, and most learning health systems fall into more than one category. |
| **Phase of implementation** | **Planning:** currently in development, and no evidence that it is active.  **Active:** describes when the learning health system was initiated and / or describes learning activities that have been done or are currently be done; no evidence that learning health system is no longer active.  **Dormant**:** describes that funding has ended or the learning health system is currently inactive for another reason, but with plans to continue in the future.  **We also had a category on ‘completed’ learning health systems, but the only ones we identified where they described the learning health system had ended (generally due to funding) and no evidence of it continuing in the future were networks with potential to be learning health systems, and did not meet our definition of true learning health systems. We decided to exclude these from the potential learning health systems table. |
| **Likelihood of future sustainability** | Whether the funding sources were external and time-limited (e.g., research grants for a certain time period), or if it was using internal funding and resources without a time restraint. In some cases, sustainability of the learning health system was described in the data source. |
| **Whether they call themselves a learning health system** | If LHS was identified as part of the name of the network, in the title of the data source, or within the body of the data source. |
| **Challenges and strengths** | Description of specific challenges or strengths encountered or anticipated for the development of the LHS itself or the learning activities described in the data source. |
| **Example projects / learning activities** | Description of projects or learning activities completed through the LHS; i.e. either research projects or quality improvement initiatives, depending on their priorities. |
