# Supplementary material for: Learning health systems in primary care: a systematic scoping review
Source: BMC Fam Pract. 2021 Jun 23;22:126. doi: 10.1186/s12875-021-01483-z (PMC8223335; doi:10.1186/s12875-021-01483-z)
Supplement: Supplementary file 4 — Additional file 4. Integrated health systems and data networks with potential to become learning health systems. Summary of the identified integrated health systems and data networks with potential to become learning health systems and explanation for why they were not considered fully-functioning learning health systems [file 12875_2021_1483_MOESM4_ESM.docx]

**Additional File 4. Integrated health systems and data networks with potential to become learning health systems**

| **Name** | **Location** | **Description** | **Explanation for Exclusion** |
| --- | --- | --- | --- |
| **Integrated health system** | | | |
| PCORnet – HealthCore^1^ | U.S. | The research branch of Anthem Inc. health benefits company. Includes health plan data, including primary care data from across 14 States. | Seems like it is just a research organization and not a true LHS. Describes itself as a service provider to researchers, government, etc. Disparate research studies are conducted rather than this information being used to feedback to practice. |
| PCORnet – PRACnet (Practice Research Network)^2^ | U.S. | Includes data from Humana Health Plans covering 27 million older Americans, including primary care data. | Seems to be more of a research network rather than a true LHS. “Practice Research Network (PRACnet) is a multi-organizational research initiative with the primary objective of conducting, promoting and facilitating meaningful patient centered research with a goal to improve the practice of healthcare”. |
| **Data network** | | | |
| CHARN (Community Health Applied Research Network)^3–6^ | U.S. | A network of Community Health Centers and academic researchers with 4 research nodes and a data coordinating center. | Seems more than just a data warehouse. The different centers work together to coordinate data compatibility and share lessons learned and different tools with one another. Yet, does not describe data being used to impact care directly. |
| Mental Health Research Network^7,8^ | U.S. | A network consisting of 13 health systems across 15 states, including Baylor Scott & White, Kaiser Permanente and Henry Ford Health System; and includes a virtual data warehouse. | It is not clear if this a true LHS, since the research results may be somewhat removed from being fed-back into practice, but it does seem like many of the researchers involved are clinicians and there are 'scientific working and interest groups' where many of this information is shared. |
| PCORnet – ADVANCE (The Accelerating Data Value Across a National Community Health Center)^9^ | U.S. | Outpatient EHR data for community health center patients, including hospital and health plan data for these patients across 26 different States. | Seems mostly like a data / research network with patient engagement efforts. Mentions promoting the individual centres to be LHS but does not describe itself as one. “The goal of ADVANCE is to build and maintain a “community laboratory” of Federally Qualified Health Centers (FQHCs) serving safety net patients, including the uninsured, the under-insured, undocumented immigrants, and other vulnerable populations.” |
| PCORnet – CAPriCORN (Chicago Area Patient-Centered Outcomes Research Network)^10,11^ | Chicago, U.S. | A network consisting of 11 healthcare partners including hospitals (including two VA hospitals) and health systems including primary care. | Seems like it is just a data platform for research. Does not talk about using this data to feedback and improve care at all. “CAPriCORN will establish a network to share data, identify a cohort of over one million patients in the Chicago area, and establish a sustainable platform for patient-centered outcomes research that engages clinicians, patients, and other stakeholders in carrying out meaningful research.” |
| PCORnet – GPC (Greater Plains Collaborative)^12,13^ | U.S. | A network consisting of 10 medical centers including hospitals and clinics (mostly for specialist care but also some primary care) across 7 states. | Seems more like a data network and not a real LHS. Only focuses on the data, and research using the data but does not provide any specific examples of how this information can feedback to improve clinical care. “GPC is a network of 12 leading medical centers in 9 states committed to a shared vision of improving healthcare delivery through ongoing learning, adoption of evidence - based practices, and active research dissemination”. |
| PCORnet – OneFlorida^14,15^ | Florida, U.S. | State-wide network collaboration between the University of Florida Clinical and Translational Science Institute, Florida State University, and the University of Miami, and includes 8 health systems (including primary care). | Seems to mostly be a research network. Does discuss more pragmatic trials and not just observational research, but does not mention how this research will feedback to improve care of patients. Does mention the following: “Over time, the consortium envisions creating a learning network for the state, in which lessons from research and care in diverse settings are systematically captured and translated back into improved health, health care and health policy for Floridians”. |
| PCORnet – REACHnet (Research Action for Health Network)^16^ | U.S. | Collaboration between Louisiana Public Health Institute, Ochsner Health System, Partnership for Achieving Total Health (PATH), Louisiana State University, Pennington Biomedical Research Center, Tulane University, and Baylor Scott & White Health. Includes primary data through healthcare plans including Humana, Blue Cross Blue Shield and Louisiana Medical. | Seems to be more of a research network rather than a true LHS. "Our mission is to facilitate research that addresses healthcare questions of critical importance to patients and clinicians and contributes to the evidence base that will inform more effective healthcare decision-making and improve population health". |
| PCORnet – SCILHS (Scalable Collaborative Infrastructure for a Learning Health System)^17,18^ * | U.S. | Network includes 10 health systems: Beth Israel Deaconess Medical Center, Boston Children's Hospital, Boston Health Net, Cincinnati Children's Medical Center, Columbia University Medical Center & New York Presbyterian Hospital, Morehouse School of Medicine/Grady Memorial Hospital, Partners HealthCare System, University Mississippi Medical Center, University of Texas Health Science Center at Houston, and Wake Forest Baptist Medical Center. | Not clear if this is a true LHS, since it is essentially a data network like many of the other PCORI-funded projects, but it does describe providing support using innovative apps at the point of care. |
| PCORnet – STAR (Stakeholders, Technology and Research)^19^ | U.S. | Includes academic health centers, community hospitals and outpatient practices including primary care across Tennessee, North Carolina, South Carolina, and Minnesota. | Not clear if this is true LHS. Seems mostly like a data network like the other PCORI-funded projects, but does describe use of informatics tools and research that focuses on health system innovation. However, it does not provide any specific examples of this and there is no reference to it as an LHS. “The objective of our CRN is to robustly support comparative effectiveness studies, pragmatic clinical trials, health system innovation, and the other research needs of our stakeholders and partners”. |
| SMASH (Salford Medication Safety Dashboard)^20–22^ | Salford, U.K. | A large urban area consisting of one hospital and 45 primary care practices. | Not clear if this is true LHS. Although the use and evaluation (in a pragmatic trial) of an EHR feedback tool for inappropriate prescribing seems like an LHS use case, it is not clear how this is different from other pragmatic trials evaluating a similar intervention. One of the investigators confirmed through email communication that this was the only intervention using this platform that he is aware of. It was decided that this is not a true LHS. |
| **Network of networks** | | | |
| PCORnet – NYC-CDRN (New York City Clinical Data Research Network)^23,24^ | New York, U.S. | Collaboration of 22 organizations, including 7 independent health systems and primary care centers. | Seems like a research data network and not a true LHS. Describes the data, patient engagement, support for multi-center trials, but does not describe how the data is used to feedback and improve care. “The INSIGHT Clinical Research Network (INSIGHT CRN) has been established to improve and streamline research in an effort to advance patient-centered research”. |
| PCORnet – PaTH^25,26^ | Mid-Atlantic region, U.S. | Network includes community hospitals, academic institutes, and outpatient practices that provide healthcare to a population of 2.5 million patients, including Geisinger and Johns Hopkins University. | Seems to be more of a research network focused on patient engagement. Does not describe any specific examples of how information can be fedback to improve care. “PaTH utilizes Patient Empowered Research to address the questions and concerns that matter most to our communities in order to make better health decisions”. |
| PCORnet – pSCANNER (Scalable National Network for Effectiveness Research)^27,28^ * | U.S. | Network includes three large existing health systems including 13 total data sites (where some include primary care clinics): Veteran Health Administration (inpatient, ambulatory and outpatient clinics), the University of California Research exchange network, and a University of Southern California led initiative called SCANNER. | Seems to be more of a data network rather than a true LHS. “Designed to be a stakeholder-governed federated network that uses a distributed architecture to integrate data from three existing networks”. |
| PORTAL (Kaiser Permanente & Strategic Partners Patient Outcomes Research To Advance Learning)^29,30^ | U.S. | PORTAL links 4 integrated health systems (including primary care services): Kaiser Permanente, Group Health Cooperative, HealthPartners, and Denver Health. | Seems to be more of a research network rather than a true LHS. “PORTAL network offers a robust and experienced platform for comparative effectiveness and patient-centered outcomes research”. |

*These networks are currently dormant.

Abbreviations: Learning Health System, LHS; National Patient-Centered Clinical Research Network, PCORnet

**References**

1. HealthCore. HealthCore. https://www.healthcore.com/. Published 2019. Accessed November 21, 2019.

2. PRACnet. Practice Research Network (PRACnet). https://www.pracnet.org/. Published 2019. Accessed November 21, 2019.

3. Laws R, Gillespie S, Puro J, et al. The Community Health Applied Research Network (CHARN) Data Warehouse: a Resource for Patient-Centered Outcomes Research and Quality Improvement in Underserved, Safety Net Populations. *eGEMs (Generating Evid Methods to Improv patient outcomes)*. 2014;2(3):11. doi:10.13063/2327-9214.1097

4. Likumahuwa S, Song H, Singal R, et al. Building Research Infrastructure in Community Health Centers: A Community Health Applied Research Network (CHARN) Report. *J Am Board Fam Med*. 2013;26(5):579-587.

5. Vargas N, Lebrun-Harris LA, Weinberg J, Dievler A, Felix KL. Qualitative Perspective on the Learning Health System: How the Community Health Applied Research Network Paved the Way for Research in Safety-Net Settings. *Prog Community Heal Partnerships Res Educ Action*. 2018;12(3):329-339. doi:10.1353/cpr.2018.0057

6. Community Health Applied Research Network. CHARN. https://www.kpchr.org/CHARN/public/index.aspx?pageid=1. Published 2013. Accessed November 21, 2019.

7. Rossom RC, Simon GE, Beck A, et al. Facilitating Action for Suicide Prevention by Learning Health Care Systems. *Psychiatr Serv*. 2016;67(8):830-832. doi:10.1176/appi.ps.201600068

8. Mental Health Research Network. Mental Health Research Network. http://hcsrn.org/mhrn/en/. Published 2019. Accessed November 21, 2019.

9. ADVANCE Collaborative. ADVANCE Collaborative. http://advancecollaborative.org/. Published 2019. Accessed November 21, 2019.

10. Kho AN, Hynes DM, Goel S, et al. CAPriCORN: Chicago area patient-centered outcomes research network. *J Am Med Informatics Assoc*. 2014;21(4):607-611. doi:10.1136/amiajnl-2014-002827

11. Chicago Area Patient-Centered Outcomes Research Network. CAPriCORN. http://capricorncdrn.org/. Published 2019. Accessed November 21, 2019.

12. Waitman LR, Aaronson LS, Nadkarni PM, Connolly DW, Campbell JR. The greater plains collaborative: A PCORnet clinical research data network. *J Am Med Informatics Assoc*. 2014;21(4):637-641. doi:10.1136/amiajnl-2014-002756

13. Greater Plains Collaborative. Greater Plains Collaborative. http://www.gpcnetwork.org/. Published 2019. Accessed November 21, 2019.

14. Shenkman E, Hurt M, Hogan W, et al. OneFlorida clinical research consortium: Linking a clinical and translational science institute with a community-based distributive medical education model. *Acad Med*. 2018;93(3):451-455. doi:10.1097/ACM.0000000000002029

15. OneFlorida Consortium. OneFlorida. https://onefloridaconsortium.org. Published 2019. Accessed November 21, 2019.

16. REACHnet. Research Action for Health Network. reachnet.org. Published 2019. Accessed November 21, 2019.

17. Mandl KD, Kohane IS, McFadden D, et al. Scalable collaborative infrastructure for a learning healthcare system (SCILHS): Architecture. *J Am Med Informatics Assoc*. 2014;21(4):615-620. doi:10.1136/amiajnl-2014-002727

18. Patient-Centered Outcomes Research Institute. Scalable Collaborative Infrastructure for a Learning Healthcare System (SCILHS) - Phase I. https://www.pcori.org/research-results/2013/scalable-collaborative-infrastructure-learning-healthcare-system-scilhs-phase. Published 2019. Accessed November 21, 2019.

19. Stakeholders Technology and Research CRN. STAR. https://starcrn.org/. Published 2019. Accessed November 21, 2019.

20. Jeffries M, Keers RN, Phipps DL, et al. Developing a learning health system: Insights from a qualitative process evaluation of a pharmacist-led electronic audit and feedback intervention to improve medication safety in primary care. *PLoS One*. 2018;13(10). doi:10.1371/journal.pone.0205419

21. Williams R, Keers R, Guide WT, et al. SMASH! The Salford medication safety dashboard. *J Innov Heal Informatics*. 2018;25(3):183-193.

22. Health e-Research Centre. The Salford Medication Safety Dashboard. https://www.herc.ac.uk/case_studies/salford-medication-safety-dashboard/. Published 2019. Accessed November 22, 2019.

23. Kaushal R, Hripcsak G, Ascheim DD, et al. Changing the research landscape: The New York City clinical data research network. *J Am Med Informatics Assoc*. 2014;21(4):587-590. doi:10.1136/amiajnl-2014-002764

24. New York City Clinical Data Research Network. NYC-CDRN. http://www.nyccdrn.org/. Published 2015. Accessed November 21, 2019.

25. Amin W, Tsui F, Borromeo C, et al. PaTH: Towards a learning health system in the Mid-Atlantic region. *J Am Med Informatics Assoc*. 2014;21(4):633-636. doi:10.1136/amiajnl-2014-002759

26. PaTH Network. Welcome to the PaTH Clinical Research Network. http://pathnetwork.org/. Published 2016. Accessed November 21, 2019.

27. Ohno-Machado L, Agha Z, Bell DS, et al. pSCANNER: Patient-centered Scalable National Network for Effectiveness Research. *J Am Med Informatics Assoc*. 2014;21(4):621-626. doi:10.1136/amiajnl-2014-002751

28. pSCANNER. Patient-Centered Scalable National Network for Effectiveness Research. http://pscanner.ucsd.edu/. Published 2017. Accessed November 21, 2019.

29. McGlynn EA, Lieu TA, Durham ML, et al. Developing a data infrastructure for a learning health system: The PORTAL network. *J Am Med Informatics Assoc*. 2014;21(4):596-601. doi:10.1136/amiajnl-2014-002746

30. Patient-Centered Outcomes Research Institute. Kaiser Permanente & Strategic Partners Patient Outcomes Research To Advance Learning (PORTAL) Network - Phase I. https://www.pcori.org/research-results/2013/kaiser-permanente-strategic-partners-patient-outcomes-research-advance. Published 2019. Accessed November 21, 2019.
